# Development of an Intervention Setting Ontology for behaviour change: Specifying where interventions take place

**DOI:** 10.12688/wellcomeopenres.15904.1

**Published:** 2020-06-10

**Authors:** Emma Norris, Marta M. Marques, Ailbhe N. Finnerty, Alison J. Wright, Robert West, Janna Hastings, Poppy Williams, Rachel N. Carey, Michael P. Kelly, Marie Johnston, Susan Michie

**Affiliations:** 1 Centre for Behaviour Change, University College London, London, UK; 2 Department of Clinical Sciences, Brunel University, Uxbridge, UK; 3 ADAPT SFI Research Centre, Trinity College Dublin, Dublin, Ireland; 4 Research Department of Epidemiology & Public Health, University College London, London, UK; 5 Primary Care Unit, Institute of Public Health, University of Cambridge, Cambridge, UK; 6 Aberdeen Health Psychology Group, University of Aberdeen, Aberdeen, UK

**Keywords:** ontology, behaviour change, context, evidence synthesis, intervention reporting, stakeholder review

## Abstract

**Background**: Contextual factors such as an intervention’s setting are key to understanding how interventions to change behaviour have their effects and patterns of generalisation across contexts. The intervention’s setting is not consistently reported in published reports of evaluations. Using ontologies to specify and classify intervention setting characteristics enables clear and reproducible reporting, thus aiding replication, implementation and evidence synthesis. This paper reports the development of a Setting Ontology for behaviour change interventions as part of a Behaviour Change Intervention Ontology, currently being developed in the Wellcome Trust funded Human Behaviour-Change Project.

**Methods**: The Intervention Setting Ontology was developed following methods for ontology development used in the Human Behaviour-Change Project: 1) Defining the ontology’s scope, 2) Identifying key entities by reviewing existing classification systems (top-down) and 100 published behaviour change intervention reports (bottom-up), 3) Refining the preliminary ontology by literature annotation of 100 reports, 4) Stakeholder reviewing by 23 behavioural science and public health experts to refine the ontology, 5) Assessing inter-rater reliability of using the ontology by two annotators familiar with the ontology and two annotators unfamiliar with it, 6) Specifying ontological relationships between setting entities and 7) Making the Intervention Setting Ontology machine-readable using Web Ontology Language (OWL) and publishing online.

**Re** **sults:** The Intervention Setting Ontology consists of 72 entities structured hierarchically with two upper-level classes: *Physical setting* including *Geographic location*, *Attribute of location* (including *Area social and economic condition*, *Population and resource density sub-levels*) and *Intervention site* (including *Facility, Transportation* and *Outdoor environment* sub-levels), as well as *Social setting*. Inter-rater reliability was found to be 0.73 (good) for those familiar with the ontology and 0.61 (acceptable) for those unfamiliar with it.

**Conclusion:** The Intervention Setting Ontology can be used to code information from diverse sources, annotate the setting characteristics of existing intervention evaluation reports and guide future reporting.

## Introduction

Effects of interventions to improve health vary considerably across contexts of settings and target populations. While this is widely acknowledged in the literature, the specific elements in the context and their mechanisms of action on outcomes are either assumed or obscure ( Michie *et al.*, 2017). In order to understand this variation arising from the different aspects of context, it is helpful to synthesise evidence about the ways in which these modifying variables influence intervention effectiveness. This requires detailed and consistent specification of study contexts. There are many different classification systems and ontologies describing interventions, including their settings and target populations; however, these have limitations such as incomplete coverage and relevance across the range of international contexts. In this paper, we consider intervention setting. A forthcoming paper will report the development of an Intervention Population Ontology ( Finnerty *et al.*, In preparation).

Intervention settings are not currently consistently reported with enough specificity or comprehensiveness to allow accurate replication. The CONsolidated Standards of Reporting Trials statement (CONSORT; Schulz *et al.*, 2010) includes one item referring to setting ( *Item 4b – Settings and locations where the data were collected*), with its extension for social and psychology interventions CONSORT-SPI ( Montgomery *et al.*, 2018) adding an additional item ( *Item 4b – Where applicable, eligibility criteria for settings and those delivering the intervention*). The Template for Intervention Description and Replication checklist (TIDieR; Hoffman *et al.*, 2014) includes one item for setting ( *Item 7 – Where: describe the type(s) of location(s) where the intervention occurred, including any necessary infrastructure or relevant features*). The recent Typology of Interventions in Proximal Physical Micro-Environments typology (TIPPME: Hollands *et al.*, 2017) allows specification of micro-level aspects of the physical environment related to behaviours. Although this was based on an exhaustive review of the literature, TIPPME is restricted to interventions in micro-environments or contexts aimed at changing selection, purchase and consumption of food, alcohol and tobacco. We currently lack a classification system to aid researchers in describing in detail, and using shared language, the variety of settings of behaviour change interventions (BCIs) or indeed behaviour more broadly.

What at first sight would seem to be a fairly straightforward task of describing intervention settings is actually very complex, given the diversity of entities, terms and definitions across academic disciplines, employment sectors and cultures. ***Ontologies*** are a tool for addressing this diversity by enabling ‘semantic inter-operability’ by associating computational data with unambiguous shared meaning ( Hastings, 2017; Michal *et al.*, 2012). Ontologies are data structures that enable precise specification of knowledge in a given domain ( Arp *et al.*, 2015). In information science, ontologies provide a set of: i) unique and unambiguous identifiers representing types of ***entity*** (such as objects, attributes or processes), ii) labels and definitions corresponding to these identifiers, and iii) specified relationships between the entities ( Arp *et al.*, 2015; Larsen *et al.*, 2017; Norris *et al.*, 2019). These labels, definitions and relationships comprise a ‘controlled vocabulary’ and formal specification for the given domain. Ontologies are dynamic representations that are maintained and updated according to new evidence about entities and relationships ( He *et al.*, 2018). Machine-readable ontologies provide an excellent structure for annotating scientific reports to allow evidence synthesis ( Michie & Johnston, 2017). As seen in other fields such as genetics ( Ashburner *et al.*, 2000), the availability and use of ontologies allows an active, iteratively developed basis for shared knowledge and understanding ( Michie & Johnston, 2017). As machine-readable artefacts, ontologies can be harnessed for ***annotation*** and evidence synthesis, such as the automation of literature searching, statistical analysis workflows and database searching and browsing, as well as in other computational applications ( Hastings, 2017) (see glossary of italicised terms in Table 1).

**Table 1.** Glossary.

Term	Definition	Source
**Annotation**	Process of coding selected parts of documents or other resources to identify the presence of ontology entities	Michie *et al.*, 2017.
**Annotation guidance** **manual**	Written guidance on how to identify and tag pieces of text from intervention evaluation reports with specific codes relating to entities in the ontology, using EPPI-Reviewer software.	
**Basic Formal Ontology** **(BFO)**	An upper level ontology consisting of continuants and occurrents developed to support integration, especially of data obtained through scientific research.	Arp *et al.*, 2015.
**Entity**	Anything that exists, that can be a continuant or an occurrent as defined in the Basic Formal Ontology.	Arp *et al.*, 2015.
**EPPI-Reviewer**	A web-based software program for managing and analysing data in all types of systematic review (meta-analysis, framework synthesis, thematic synthesis etc. It manages references, stores PDF files and facilitates qualitative and quantitative analyses such as meta-analysis and thematic synthesis. It also has a facilitate to annotate published papers.	Thomas & Brunton, 2010. *EPPI-Reviewer 4: * *http://eppi.ioe.ac.uk/eppireviewer4/* *EPPI-Reviewer Web Version: * *https://eppi.ioe.ac.uk/eppireviewer-web/*
**GitHub**	A web-based platform used as a repository for sharing code, allowing version control.	*https://github.com/*
**Inter-rater reliability**	Statistical assessment of similarity and dissimilarity of coding between two or more coders. If inter-rater reliability is high this suggests that ontology entity definitions and labels are being interpreted similarly by the coders.	Gwet, 2014. *Handbook of inter-rater reliability: The definitive guide* *to measuring the extent of agreement among raters*. Gaithersburg, Advanced Analytics.
**Interoperability**	Ontology developers should collaborate with others wherever possible to re-use entities and limit duplication of work. Interoperability of ontologies sits within the OBO Foundry principle of Commitment to Collaboration.	http://www.obofoundry.org/principles/fp-010-collaboration.html
**Issue tracker**	An online log for problems identified by users accessing and using an ontology.	BCIO Issue Tracker: https://github.com/ HumanBehaviourChangeProject/ontologies/issues
**OBO Foundry**	The Open Biological and Biomedical Ontology (OBO) Foundry is a collective of ontology developers that are committed to collaboration and adherence to shared principles. The mission of the OBO Foundry is to develop a family of interoperable ontologies that are both logically well-formed and scientifically accurate.	Smith *et al.*, 2007; *www.obofoundry.org/*
**Ontology**	A standardised representational framework providing a set of terms for the consistent description (or “annotation” or “tagging”) of data and information across disciplinary and research community boundaries.	Arp *et al.*, 2015.
**Parent class**	A subsuming class within an ontology that is related to one or more child (subsumed) classes.	Arp *et al.*, 2015.
**ROBOT**	An automated command line tool for ontology workflows.	Jackson *et al.*, 2019; http://robot.obolibrary.org
**URI**	A string of characters that unambiguously identifies an ontology or an individual entity within an ontology. Having URI identifiers is one of the OBO Foundry principles.	http://www.obofoundry.org/principles/fp-003-uris.html
**Web Ontology** **Language (OWL)**	A formal language for describing ontologies. It provides methods to model classes of “things”, how they relate to each other and the properties they have. OWL is designed to be interpreted by computer programs and is extensively used in the Semantic Web where rich knowledge about web documents and the relationships between them are represented using OWL syntax.	https://www.w3.org/TR/owl2-quick-reference/

As yet, no ontology exists to describe the complexity of behaviour change intervention settings ( Norris *et al.*, 2019). A comprehensive Behaviour Change Intervention Ontology (BCIO) is being developed as part of the Human Behaviour-Change Project ( Michie *et al.*, 2017). The BCIO consists of an upper level with 42 entities, one of which is *Behaviour change intervention setting*, specified as part of the Context in a given BCI scenario ( Michie *et al.*, 2020). Drawing on the methodology used to develop a taxonomy of behaviour change techniques (BCTTv1; Michie *et al.*, 2017) and other relevant ontologies ( Norris *et al.*, 2019), the current study aimed to develop an ontology for specifying and classifying characteristics of the settings in which interventions take place. These settings are generally applicable beyond the scope of behaviour change interventions. This paper reports the development and final version of the Intervention Setting Ontology.

## Methods

The Intervention Setting Ontology was developed in an iterative process of seven steps ( Wright *et al.*, 2020).

### Step 1 – Defining the scope of the Intervention Setting Ontology

A definition and overall topic for the ontology was set by reviewing dictionaries and the reporting guidelines of CONSORT ( Schulz *et al.*, 2010), CONSORT-SPI ( Montgomery *et al.*, 2018), TIDieR ( Hoffmann *et al.*, 2014) and TIPPME ( Hollands *et al.*, 2017).

### Step 2 – Identifying key entities and developing the preliminary Intervention Setting Ontology

An initial prototype version of the ontology was developed using both a bottom-up and top-down approach. In the bottom-up approach, 100 published reports of BCIs were reviewed to develop an initial list of intervention setting characteristics. These reports were randomly selected from a larger dataset of BCI reports partially annotated for behaviour change techniques, mechanisms of action, and modes of delivery, covering a range of health behaviours ( Carey *et al.*, 2019; Michie *et al.*, 2015).

In the top-down approach, existing classification systems of intervention setting characteristics were identified from: i) published ontologies containing terms related to behaviour change intervention setting via the Ontology Lookup Service and BioPortal; ii) the Patient, Intervention, Comparison, Outcome (‘PICO’) ontology developed by the Cochrane Collaboration due to its relevance for intervention trials; and iii) controlled medical vocabularies (e.g. SNOMED CT, MedDRA, MeSH).

The preliminary ontology contained a label and definition for each entity representing an intervention setting characteristic. Definitions were developed using pre-specified guidance, with the standard format of definitions being: A is a B that C, or involves or relates to C in some way, where A is the class being defined, B is a ***parent class*** and C describes a set of properties of A that distinguish it from other members of B ( Michie *et al.*, 2019). It was piloted with published BCI reports focusing on smoking cessation and physical activity behaviours ( Michie *et al.*, 2017). BCI reports were annotated independently by two researchers in batches of 10, with each entity annotated as either present or absent. Two types of inter-rater reliability measures were used: i. percentage of agreement between coders and ii. Cohen’s Kappa ( Cohen, 1960). Kappa statistics are only reported in instances where the researchers allocated a code to at least five cases ( Michie *et al.*, 2015). Satisfactory inter-rater reliability was achieved by the time 55 papers had been coded. After this, no additional adjustments were made to the prototype version of the Intervention Setting Ontology.

### Step 3 – Refinement of the ontology through literature annotation, discussion and revision

The preliminary ontology was revised by the research team based on the results of the pilot annotations. Using ***EPPI-Reviewer*** 4 software ( Thomas & Brunton, 2010), two researchers independently annotated 30 BCI reports on smoking cessation interventions using the revised Intervention Setting Ontology. An open alternative to this software used for annotation is PDFAnno ( Shindo *et al.*, 2018). Discrepancies were discussed and the ontology structure, definitions and ***annotation guidance manual*** were revised. A second set of annotators followed the same procedure for another set of 45 BCI reports of smoking cessation, and 40 BCI reports of physical activity. All reports were randomised controlled trials from one of three datasets: Cochrane Reviews, papers annotated for behaviour change techniques and papers from the IC-SMOKE project ( Black *et al.*, 2020; De Bruin *et al.*, 2016) (List of papers used in development of ontology: https://osf.io/4qcby/ ( West *et al.*, 2020)).

### Step 4 – Expert stakeholder review

Ninety-eight members of a panel of behavioural scientists and public health expert stakeholders were invited to give feedback on the Intervention Setting Ontology resulting from Step 3. These experts comprised i) 65 behavioural scientists who had provided feedback on previous projects at the Centre for Behaviour Change, ii) 16 experts from under-represented countries identified through the BCTTv1 database, and iii) 17 stakeholders who expressed interest in being involved in the Human Behaviour-Change Project stakeholder initiatives. Experts from both 'well-represented' countries (UK, USA, Canada, Australia, the Netherlands) and other 'less-represented' countries were randomly selected to provide feedback using Researcher Randomizer.

Feedback was collected through an online questionnaire, using Qualtrics ^TM^ software (Full survey https://osf.io/8audy/ ( West *et al.*, 2020)), with the task designed to take no longer than 45 minutes to complete. The task asked experts to:

1. identify the characteristics of intervention setting that were of *interest* to them when trying to understand variation in the effectiveness of BCIs (open-ended question). Experts were advised to consider a specific behaviour when answering this question e.g ‘physical activity’
2. rate the *importance* of each of the setting entities on a 5-point Likert scale (1 = “not important”, 2 = “slightly important”, 3 = “moderately important”, 4 = “important”, 5 = “very important” or “don’t know/not sure”). For example: “How *important* do you think each of the following Geographic location characteristics are to understand variation in the effectiveness of at least some behaviour change interventions?” (Country of intervention & Within country location), and
3. provide feedback on the *completeness* and *comprehensiveness* of the Setting Ontology.

Experts were also asked to indicate: i) if there were any entities missing (If yes, which should be added), ii) if there were any entities or definitions that should be changed (if yes, what changes should be considered), and iii) If there were any entities that should be placed in a different location in the classification hierarchy of the Intervention Setting Ontology.

A thematic analysis of the responses was conducted and means and standard deviations of ratings were calculated. The feedback from the expert consultation was discussed by the research team and the Intervention Setting Ontology and annotation guidance were revised.

### Step 5 – Inter-rater Reliability of Annotations using the Intervention Setting Ontology

Assessment of ***inter-rater reliability*** of the annotations by two researchers leading the development of the ontology was conducted using 50 papers from Cochrane reviews (30 for smoking cessation and 20 for physical activity). Inter-rater reliability was also assessed for annotations by two behaviour change experts unfamiliar with the ontology but with experience in annotating BCI reports. Annotation was of a random sample of 50 randomised controlled trials from a database of papers coded by Behaviour Change Techniques, with no restrictions on the outcome behaviour. Inter-rater reliability was assessed using Krippendorff’s Alpha ( Hayes & Krippendorff, 2007) using Python 3.6 ( https://github.com/HumanBehaviourChangeProject/Automation-InterRater-Reliability) ( Finnerty & Moore, 2020), as unlike Cohen’s Kappa, Krippendorff factors in both agreement and disagreement within annotations.

### Step 6 – Specifying the relationships between Intervention Setting Ontology entities

The research team established relationships between ontology entities to formalise the knowledge present in the ontology. This process was conducted in line with ***Basic Formal Ontology*** principles which have been used extensively in biomedical ontologies ( Arp *et al.*, 2015). The suitability of common relationships from Basic Formal Ontology ( Arp *et al.*, 2015) and the Relation Ontology ( Smith *et al.*, 2005) were assessed, including the basic hierarchical relationship ‘ *is_a’* which holds between classes where one class is a subclass of another class *,* and ‘ *located_in’*, which relates an entity to a spatial region demarcating a location.

### Step 7 - Making the Intervention Setting Ontology machine-readable and available online

The Intervention Setting Ontology was initially developed as a table of entities, with separate rows for each entity annotated with a primary label, definition, synonyms and relationships. When the Intervention Setting Ontology was at a stable level of development for initial release, it was converted into the ***Web Ontology Language (OWL)*** ( Antoniou & Van Harmelen, 2004) format, enabling it to be viewed and visualised using ontology software such as Protégé and to be compatible with other ontologies. The conversion to OWL used the ***ROBOT*** ontology toolkit library ( Jackson *et al.*, 2019), which provides a facility to create well-formatted ontologies from templates. A ROBOT template is a comma-separated values (CSV) file that can be prepared easily in common spreadsheet software, annotated with instructions for translation from spreadsheet columns to OWL language and metadata attributes. Within the input template spreadsheet, separate columns represent the entity ID (e.g. BCIO_0013), name, definition, relationship with other entities, examples and synonyms.

This OWL version of the Intervention Setting Ontology was then stored on the project ***GitHub*** repository, as GitHub has an ***issue tracker*** which allows feedback to be submitted by members of the community which can be responded to, and if necessary, addressed in subsequent releases. When the full Behaviour Change Intervention Ontology has been confirmed, it will be submitted to the ***OBO Foundry*** ( Smith *et al.*, 2007).

## Results

### Step 1 – Defining the scope of the Intervention Setting Ontology

Given that ‘setting’ is defined in a general lexicon as ‘ *the place or type of surroundings where something is positioned or where an event takes place*’, an intervention’s setting was defined more precisely as ‘ *An aggregate of entities that form the environment in which a BCI is provided*.’

### Step 2 - Identifying key entities and developing the preliminary Intervention Setting Ontology

The initial prototype version of the Intervention Setting Ontology encompassed a four-level hierarchical structure, containing 76 unique entities ( https://osf.io/g8qfv/ ( West *et al.*, 2020)). Inter-rater agreement for identifying the presence of a setting entity was low in terms of percentage, at 45.5%. Kappa statistics varied from ‘perfect’ for entities such as *Accommodation* to low agreement (κ=0.300) for entities such as *Community setting*.

### Step 3 – Refinement of the Intervention Setting Ontology

Based on the annotations from Step 2, changes were made to the ontology. Two terms, ‘particular‘ and ’unclear/not reported‘, were deleted as they did not meet the ontological requirement of being unique discrete entities with corresponding definitions and attributes ( Arp *et al.*, 2015). Other changes were:

1) *Health Care facility* was revised from having the lower-level entities *Primary care, Secondary Care, Tertiary Care, Pharmacy and Hospice*, to having lower-level entities of *Hospital facility, Doctor-led primary care facility, Care home facility, Hospice facility, Psychiatric facility, Pharmacy facility, Community health care facility and Dentist facility*;

2) *Public transportation* was extended from only public transportation to a new entity named *Transportation* which includes *Public transportation, Mobile intervention venue* as well as *Private transportation*;

3) *Outdoor environment* was added to the ontology;

4) *Attribute of location* was added to the ontology, including new entities *Area social and economic condition* and *Population and resource distribution* (previously placed in Geographic location). Changes to labels and definitions were made to reflect the structural changes.

### Step 4 – Expert stakeholder review

Of the 98 experts contacted, 78 were from ‘well-represented’ countries and 20 from ‘less-represented’ countries. Of the 23 experts (23.5%) completing the survey, 19 were from ‘well-represented’ and four from ‘less-represented’ countries. Experts’ responses and how these were addressed within the ontology development are reported at: https://osf.io/npsy7/ ( West *et al.*, 2020).

The setting entities rated as of top importance by experts were *Area social and economic condition* (M=4.28/5; SD=0.87), *Outdoor environment* (M=4.28; SD=1.24), *Healthcare facility* (M=4.22; SD=0.79), *Educational facility* (M=4.06; SD=0.85), *Transportation* (M=4.06; SD=1.27) and *Community facility* (M=-4.00; SD=1.00).

Changes made to the Intervention Setting Ontology as a result of stakeholder feedback included adding *Suburban area density*, *Developed-* and *Developing country* and expanding examples within *Sport and exercise facilit*y such as swimming pool and stadium. Suggestions to add eHealth or mHealth intervention descriptors (n=3) were not incorporated in the Intervention Setting Ontology, as these are classified in the Modes of Delivery ontology ( Marques *et al.*, 2020) within the wider Behaviour Change Intervention Ontology ( https://osf.io/h4sdy/ ( West *et al.*, 2020)). Some suggested changes were not made as they would have decreased the generalisability of the Intervention Setting Ontology. For example, a suggestion to add a variety of school types such as *Voluntary Aided (VA), State, Private, Faith, Academies* etc would have led to UK-specific terminology ( UK Government, 2019). The broad approach of classifying school settings as *Primary*, *Middle* or *Secondary school* was maintained to capture the range of international school settings.

### Step 5 – Inter-rater reliability of annotations using the Intervention Setting Ontology

Inter-rater reliability from the 50 papers annotated by those familiar with the ontology was found to be good ( *a*=0.73). The random selection of 50 papers used for inter-rater reliability testing in those unfamiliar with the ontology resulted in papers with the following target behaviours: physical activity (k=16), dietary behaviours (k=9), sexual behaviours (k=8), alcohol (k=7) and other behaviours such as medication adherence (k=11). The inter-reliability for these annotations was acceptable ( *a*=0.61) ( Hayes & Krippendorff, 2007).

### Step 6 – Specifying the relationships between Intervention Setting Ontology entities

Relationships from the Relation Ontology ( Smith *et al.*, 2005) were used to connect classes, namely the basic hierarchical relationship ‘ *is_a’* which holds between classes where one class is a subclass of another class, and ‘ *is_attribute_of’* which holds between classes where one class is a quality or feature of the other.

### Step 7 – Making the Intervention Setting Ontology machine-readable and available online

A downloadable version of the final Intervention Setting Ontology is available from GitHub ( Norris *et al.*, 2020). The hierarchical structure, ***URIs***, labels and definitions for all entities are described in Table 2. The ontology is accompanied by an annotation guidance manual that provides guidance on how to annotate for these entities in BCI reports (available at https://osf.io/76jty/) ( West *et al.*, 2020).

**Table 2.** Entity labels, definitions and URIs for all Intervention Setting Ontology Entities.

Upper-Level	Sub-Level 1	Sub-Level 2	Sub-Level 3	Sub-Level 4	Sub-Level 5	Definition
Physical setting BCIO:026000						A physical environment in which a BCI is delivered.
	Geographic location GAZ_00000448					A reference to a place on the Earth, by its name or by its geographical location
		Country of intervention BCIO:026001				A geographic location of a country where the intervention takes place.
		Within-country location BCIO:026002				A geographic location within a country where the intervention takes place. Example: region, town, city, state
	Attribute of location BCIO:026003					Features of a given location, such as social and economic characteristics.
		Area social and economic condition BCIO:026004				An attribute of location describing the overall socioeconomic state of a location. Example: disadvantaged area, crime rates, World Bank Classifications
			Low-income area BCIO:026005			An area social and economic condition described to have a low-income, whether at a country or within-country level. Example: developing country
			High-income area BCIO:026006			An area social and economic condition described to have a high- income, whether at a country or within-country level. Example: developed country
		Population and resource density BCIO:0026007				An attribute of location describing the density of an area, in terms of people and resources within it.
			Rural area ENVO:01000772			An area which is outside of a town, city, or urban area. Rural areas are primarily used for agriculture or pastoralism and may contain rural settlements
			Suburban area BCIO: 026008			An area on the edge of a large town or city where a proportion of those who work in the town or city live
			Urban area ENVO:01000856			Incorporated populated place
	Site BFO_0000029					A three-dimensional immaterial entity that is (partially or wholly) bounded by a material entity or it is a three-dimensional immaterial part thereof
		Facility OMRSE:00000062				An architectural structure that bears some function.
			Residential facility OMRSE_00000191			A facility that has at least one housing unit as part in which a person or persons live
				Household residence BCIO:026009		A facility where an individual is living alone or with one or more person. The individuals do not have to be related
				Multiple occupancy residence BCIO:026010		A facility where an individual lives with many others that may be collected according to a social structure such as education or disability. Example: bedsit
					Student residence BCIO:026011	A multiple occupancy residence where many students live. Example: student halls
					Residential care or assisted living BCIO:026012	A multiple occupancy residence where multiple vulnerable people live. Example: retirement home
				Homeless setting BCIO:026013		A residential facility where an individual is living that is not stable and secure. Example: an architectural structure for which an individual does not have a legal right to stay
				Temporary residence BCIO:026014		A residential facility where individuals are in a transitional state of housing and not staying for a prolonged period. Example: Hostels, B&B, emergency accommodation,
			Healthcare facility OMRSE:00000102			A facility that is administered by a health care organisation for the purpose of providing health care to a patient population
				Hospital facility OMRSE:00000063		A facility that is run by a hospital organization, such as emergency departments, outpatient clinics and rehabilitation and is the bearer of a hospital function
					Emergency department facility OMRSE:00000114	A health care facility that bears a function to provide emergency healthcare services and the acute care of patients who present without prior appointment, having arrived either by their own means or by ambulance
					Hospital outpatient clinic facility BCIO:026015	A hospital facility to treat patients without them staying overnight, often after a hospital visit
				Doctor-led primary care facility BCIO:026016		A healthcare facility led by doctors. Example: general practitioner surgery, doctors' surgery
				Care home facility BCIO:026017		A healthcare facility that is run by a care home organization and is the bearer of a care home function.
				Hospice facility OMRSE:00000104		A health care facility that bears a function to provide healthcare to the sick or terminally ill
				Pharmacy facility PDRO:0000074		A health care facility whose function is to store, prepare, dispense, and monitor the usage of pharmaceutical drugs among patients in a given area or encountered in a given health care provider organization
				Rehabilitation facility OMRSE:00000106		A facility to assist in physical or addiction recovery
					Drug or alcohol rehabilitation facility BCIO:026018	A rehabilitation facility to assist the recovery of people with drug or alcohol addiction.
				Psychiatric facility NCIT:C53536		A place designed and staffed to house and treat individuals that need assistance with mental dysfunctions
				Community healthcare facility BCIO:026019		A clinic providing healthcare services to people in a certain area. Example: polyclinic
					Community outpatient clinic facility BCIO:026020	A healthcare facility to treat patients in the community without them staying overnight.
				Dentist facility BCIO:026021		A healthcare facility where dental healthcare is provided.
			Educational facility BCIO:026022			A facility in which formal education is provided to a student population.
				Early years facility BCIO:026023		An educational facility in which pre-school education is provided. Example: nursery school
				School facility OMRSE:00000064		A facility that is run by a school organization and is the bearer of a school function
					Primary school BCIO:026024	A school facility for younger children, typically aged between five and eleven
					Middle school BCIO:026025	A school facility providing education between primary and secondary school
					Secondary school BCIO:026026	A school facility for older children and teenagers, typically aged between eleven and eighteen Example: high school
				Vocational facility BCIO:026027		An educational facility providing practically based, occupationally-specific teaching.
				University facility BCIO:026028		An educational facility in which students study for degrees and academic research is done.
			Community facility BCIO:026029			A facility used by a group of people living in the same place or having a particular characteristic in common. Example: food bank, recycling centre
				Sport and exercise facility BCIO:026030		A community facility used for exercising. Example: gym, stadium, tennis courts, swimming pool
				Social centre or Community Hall facility BCIO:026031		A community facility used for socialising by those living in a given area. Example: YMCA or working men’s club
				Library facility BCIO:026032		A community facility containing a collection of books and learning resources for loan.
				Religious facility BCIO:026033		A community facility where individuals or a group of people come to perform acts of devotion and veneration. Example: mosque, church, temple
				Hospitality and catering facility BCIO:026034		A community facility used to serve food. Example: diner, restaurant, pub or bar
				Arts and entertainment facility BCIO:026035		A community facility designed to entertain or amuse. Example: cinema, theatre, disco
			Retail facility BCIO:026036			A facility used as an outlet for shopping. Example: supermarket, market or shopping centre
			Research facility ENVO:00000469			A facility, permanent or temporary, on land, in air, space or water, where scientific research or measurements can be undertaken Example: research lab
			Office facility BCIO:026037			A facility of a room, set of rooms, or building used as a place for commercial, professional, or bureaucratic work
			Criminal justice Facility BCIO:026038			A facility where individuals are being reprimanded, detained or imprisoned Example: prison
			Factory facility BCIO:026039			A facility of a building or group of buildings where goods are manufactured or assembled chiefly by machine
			Military facility BCIO:026040			A facility relating to or characteristic of soldiers or armed forces. Example: army, navy, air force
		Transportation NCIT_C141286				Methods of traveling from one place to another.
			Public transportation NCIT:C141287			Forms of transportation that run on fixed routes and are available to the public, usually for a set fare e.g bus, train, plane
			Private transportation BCIO:026041			A form of transportation owned by an individual for individual or group use. Example: car, bicycle, motorbike
			Mobile intervention venue BCIO:026042			A form of transportation delivering interventions in transient locations. Example: mobile van
			Ambulance BCIO:026043			A form of transportation which can transport patients for health treatment, and in some instances will also provide out-of-hospital healthcare to the patient.
		Outdoor environment BCIO:026044				A site which is an outdoor location outside of a building.
			Park ENVO:00000562			A bounded area of land, or water, usually in its natural or semi-natural (landscaped) state and set aside for some purpose, usually to do with recreation or conservation
			Forest ENVO:00000111			An area with a high density of trees. A small forest may be called a wood
			Beach ENVO:00000091			A landform consisting of loose rock particles such as sand, gravel, shingle, pebbles, cobble, or even shell fragments along the shoreline of a body of water
			Water BCIO:026045			An outdoor environment set in an expanse of water.
			Grassland ENVO:00000106			An area in which grasses (Graminae) are a significant component of the vegetation
			Road ENVO:00000064			An open way for the passage of vehicles, persons, or animals on land
			Path or pavement BCIO:026046			An outdoor environment for the passage of persons or cyclists on land.
				Path or pavement for pedestrians BCIO:026047		A path or pavement for the passage of persons only on land.
				Path or pavement for cyclists BCIO:026048		A path or pavement for the passage of people using bicycles only on land.
Social setting (BCIO:029000)						An aggregate of people with whom a BCI population interacts.

**Note:** Entity URIs correspond to Behaviour Change Intervention Ontology (BCIO), Environment Ontology (ENVO), Gazetteer Ontology (GAZ), NCI Thesaurus (NCIT), Ontology of Medically Related Social Entities (OMRSE) and Prescription of Drugs Ontology (PDRO).

The final version of the Intervention Setting Ontology presents a six-level hierarchical structure comprising of 72 unique entities. There are two upper-level classes: *Physical setting* (BCIO: 026000: A physical environment in which a BCI is delivered) and *Social setting* (BCIO: 029000: An aggregate of people with whom a BCI population interacts). Physical setting includes *Geographic location (*GAZ:00000448: A reference to a place on the Earth, by its name or by its geographic location, used from the existing Gazetteer Ontology), *Attribute of location* (BCIO: 026003: Features of a given location, such as social and economic characteristics) and *Site* (BFO_0000029: A three-dimensional immaterial entity that is (partially or wholly) bounded by a material entity or it is a three-dimensional immaterial part thereof).

For each of these entities, there are lower-level entities that inherit its properties. For example *Site* includes: *Facility (*OMRSE:00000062, used from the existing Ontology of Medically Related Social Entities; Hicks *et al.*, 2016) *, Transportation* (NCIT_C141286, from the NCI Thesaurus OBO Edition; Balhoff *et al.*, 2017) and *Outdoor environment (BCIO:*026044 *)*.

Facility includes subclasses of *Residential facility* (OMRSE:00000191), *Healthcare facility* (OMRSE:00000102), *Educational facility* (BCIO:026022), *Community facility* (BCIO:026029), *Retail facility* (BCIO: 026036), *Research facility* (ENVO:00000469 from the Environment Ontology; Buttigieg *et al.*, 2013), *Office facility* (BCIO:026037), *Criminal justice facility* (BCIO:026038), *Factory facility* (BCIO:026039), and *Military facility* (BCIO:026040). Residential facility within Facility includes subclasses of *Household residence* (BCIO:026009), *Multiple occupancy residence* (BCIO:026010), *Homeless setting* (BCIO:026013) and *Temporary residence (BCIO: 026014)*. Finally, at the lowest level, Multiple occupancy residence within Residential facility has subclasses of *Student residence* (BCIO:026011) *and Residential care or assisted living* (BCIO:026012).

## Discussion

This study developed the Intervention Setting Ontology to specify formally the characteristics of the settings in which behaviour change interventions (BCIs) take place, as part of the Behaviour Change Intervention Ontology ( Michie *et al.*, 2017). Although developed primarily to specify settings of behaviour change interventions, the settings are generally applicable to other types of intervention or contexts. The ontology consists of 72 entities structured hierarchically with two upper-level classes: *Physical setting* (BCIO:026000: A physical environment in which a BCI is delivered) and *Social setting* (BCIO:029000: An aggregate of people with whom a BCI population interacts). Physical setting is further sub-divided by three upper-level classes: *Geographic location*, *Attribute of location* (including *Area social and economic*, *Population and resource density* sub-levels) and *Site* (including *Facility*, *Transportation* and *Outdoor environment* sub-levels). Inter-rater reliability was found to be 0.73 (good) for those familiar with the ontology and 0.61 (acceptable) for those unfamiliar with it, as assessed by Krippendorff’s alpha. Together with ‘population’, it makes up *Context* which is part of a wider set of lower-level ontologies within the Behaviour Change Intervention Ontology (BCIO).

The ontologies within the BCIO are connected to each other by specified relationships. For example, the contextual entity of Intervention Setting is related to the contextual entity of Population: who receives an intervention ( Finnerty *et al.*, In preparation). In addition, entities within the Intervention Setting Ontology can be integrated or linked to ontologies beyond the BCIO, a key feature of OWL ontologies which encourages re-use and adoption ( Hastings, 2017). 

Ontologies should be dynamic representations that are maintained and updated according to new evidence about entities and relationships ( Arp *et al.*, 2015; He *et al.*, 2018). The Intervention Setting Ontology and all other ontologies within the Human Behaviour-Change Project will be updated as they are informed by advances in behavioural science and by online feedback from ontology users via the GitHub portal.

### Strengths and limitations

Domain experts are often not formally consulted when ontologies are developed ( Norris *et al.*, 2019), with the result that development may be restricted to the knowledge, thinking styles and biases of individual ontology development teams. A strength of this study is the use of an explicit, standardised, tried and tested method for ontology development created within the Human Behaviour-Change Project for a range of ontologies ( Wright *et al.*, 2020). This process incorporates international expert stakeholder feedback, as has also occurred in other related projects e.g. BCTTv1, Michie *et al.*, 2015; Linking BCTs and Mechanisms of Action, Carey *et al.*, 2019; TIPPME, Hollands *et al.*, 2017; MAGI framework, Borek *et al.*, 2019. Another strength is the integration of existing terms from other ontologies where they exist, preventing duplication of entities within the wider ontology space ( Norris *et al.*, 2019). The use of entity IDs for each entity in the ontology provides a machine-readable identifier for integration in future systems and also allows ***interoperability*** between existing ontologies.

The Intervention Setting Ontology has been found to be useful to manually annotate a large body of published intervention evaluation reports ( Michie *et al.*, 2017). These manual annotations are informing the development and testing of information extraction algorithms ( Ganguly *et al.*, 2018) to automate the process of identifying and organising knowledge about interventions within published reports ( Michie *et al.*, 2020). This corpus of manually and automatically extracted data on intervention setting characteristics is being made available as it is produced on the Human Behaviour-Change Project’s GitHub page. As machine-readable representations of knowledge, these ontologies provide a framework for applying Artificial Intelligence to synthesising and interpreting evidence e.g. by identifying patterns of data organised by the BCIO. Reasoning algorithms allow real-time up-to-date evidence synthesis that can be used to answer variants of the “big question” of behaviour change: “ *What works, compared with what, for what behaviours, how well, for how long, with whom, in what setting, and why?*”, across a wide range of contexts ( Michie *et al.*, 2017). This body of work has the potential to have far-reaching use by and implications for policy-makers, practitioners and researchers, for example, by informing evidence-based guidelines, extrapolating knowledge to under-researched populations and settings, and identifying knowledge gaps.

A limitation of this work is that the intervention reports annotated within the ontology development mainly addressed two health-related behaviours, smoking cessation and physical activity. This was due to the ontology being developed within the Human Behaviour-Change Project, which is using smoking cessation and physical activity interventions as initial use cases ( Michie *et al.*, 2017). However, external inter-rater reliability was tested across diverse behaviours and found to be acceptable. Future application of the ontology to a wider collection of behaviours and contexts will help extend and improve it.

## Conclusions

The Intervention Setting Ontology provides a classification system that can be used reliably to specify the characteristics of settings where interventions take place. It will contribute to the improvement of research reporting and replication, enabling easier evidence synthesis across studies. The ontology can be used within computational tools to speed up the accumulation, interpretation and application of knowledge, such as the Knowledge System being developed within the Human Behaviour-Change Project ( Michie *et al.*, 2017). The Intervention Setting Ontology is intended to act as a foundation from which future research can build, as an ongoing and collaborative process. The ontology will allow us to increase our understanding of the settings in which interventions are implemented and how effectiveness varies across settings.

## Ethics

Ethical approval was granted by University College London’s ethics committee (CEHP/2016/555).

## Data availability

### Underlying data

The BCIO is available from: https://github.com/HumanBehaviourChangeProject/ontologies

Archived ontology as at time of publication: https://doi.org/10.5281/zenodo.3824323 ( Norris *et al.*, 2020).

License: CC-BY 4.0

### Extended data

Open Science Framework: Human Behaviour-Change Project, https://doi.org/10.17605/OSF.IO/UXWDB ( West *et al.*, 2020)

This project contains the following extended data:

- Papers used in development of the Intervention Setting Ontology.pdf (Papers used across stages of development of the Intervention Setting Ontology, with the systematic reviews that they were identified from; https://osf.io/4qcby/)
- Version 1 – Intervention Setting Ontology.pdf (Initial prototype version of Intervention Setting Ontology https://osf.io/g8qfv/)
- Setting Expert Feedback Survey.pdf (Full survey provided to behavioural science and public health experts in review of the Intervention Setting Ontology; https://osf.io/8audy/)
- Expert Feedback on Intervention Setting Ontology.pdf (Raw feedback received from behavioural science and public health experts; https://osf.io/npsy7/)
- Intervention Setting Ontology Coding Guidelines.pdf (Manual for coding using the Intervention Setting Ontology; https://osf.io/76jty/)

Data are available under the terms of the Creative Commons Attribution 4.0 International license (CC-BY 4.0).

Code used to calculate alpha for IRR: https://github.com/HumanBehaviourChangeProject/Automation-InterRater-Reliability.

Archived code as at time of publication: https://doi.org/10.5281/zenodo.3833816 ( Finnerty & Moore, 2020).

License: GNU General Public License v3.0
