# Supplementary material for: Development of an Intervention Setting Ontology for behaviour change: Specifying where interventions take place
Source: Wellcome Open Res. 2020 Jun 10;5:124. [Version 1] doi: 10.12688/wellcomeopenres.15904.1 (PMC7489274; doi:10.12688/wellcomeopenres.15904.1)
Supplement: Peer review history

# Peer review: Reviewer response for version 1

**DOI:** 10.21956/wellcomeopenres.17444.r40321

**Published:** 2020-09-11

**Authors:** Chris Noone

**Affiliations:** 1 School of Psychology, National University of Ireland Galway, Galway, Ireland

This article reports the development of an intervention setting ontology for behaviour change research. The rationale for this work is strong as the field would clearly benefit from the availability of a shared language for discussing where behaviour change interventions take place.

 This paper reports this development process very clearly and the underlying data is easy to navigate and understand. I do agree with my fellow reviewer that the article would be easier to follow if the results for each stage were reported after the methods for that stage are detailed. This would be analogous to the structure of multi-study articles.

 One important aspect of this project that could be explained in more detail is how the research community can contribute to the ongoing development of this ontology using the GitHub repository.

 For example, the term "developing country" is contested and the apparent conflation (if I have interpreted the onotology correctly) of low income countries and low income areas within countries, might be issues that researchers would like to provide feedback on, but many are not familiar with GitHub. Perhaps a guide on providing feedback could be developed and placed in the OSFproject associated with this article?

Is the work clearly and accurately presented and does it cite the current literature?

Yes

If applicable, is the statistical analysis and its interpretation appropriate?

Yes

Are all the source data underlying the results available to ensure full reproducibility?

Yes

Is the study design appropriate and is the work technically sound?

Yes

Are the conclusions drawn adequately supported by the results?

Yes

Are sufficient details of methods and analysis provided to allow replication by others?

Yes

Reviewer Expertise:

Health Psychology

I confirm that I have read this submission and believe that I have an appropriate level of expertise to confirm that it is of an acceptable scientific standard.

# Peer review: Reviewer response for version 1

**DOI:** 10.21956/wellcomeopenres.17444.r39190

**Published:** 2020-06-24

**Authors:** Tracy Epton

**Affiliations:** 1 Division of Psychology and Mental Health, School of Health Sciences, University of Manchester, Manchester, UK

The manuscript describes the development of an ontology for an intervention setting. It provides a useful tool

 There are a few minor issues that need addressing.

**Methods:**

- Step 2: For the preliminary ontology, how were the initial labels and definitions formulated once they had been extracted from the papers (bottom up approach) and existing ontologies (top down approach) e.g., process; number of people involved?

**Results:**

- Step 2: why weren’t changes made to the entities that had a low kappa to improve the reliability of rating?
- Step 4: what fields were the behavioural scientists from?

 

**Discussion:**

- Could a link to the GitHub portal be added in the text on p.7 so people can easily provide feedback on the ontology?

 

**Generally:**

- I found myself continuously going back and forth between methods and results for each step. I wonder if, in this instance, it is more appropriate to put the methods and results for each step together for ease of reading (i.e., like the reporting for a series of studies).

Is the work clearly and accurately presented and does it cite the current literature?

Yes

If applicable, is the statistical analysis and its interpretation appropriate?

Not applicable

Are all the source data underlying the results available to ensure full reproducibility?

Yes

Is the study design appropriate and is the work technically sound?

Yes

Are the conclusions drawn adequately supported by the results?

Yes

Are sufficient details of methods and analysis provided to allow replication by others?

Yes

Reviewer Expertise:

Health behaviour change

I confirm that I have read this submission and believe that I have an appropriate level of expertise to confirm that it is of an acceptable scientific standard.
